# Effectiveness and Cost-Effectiveness of Inactivated Vaccine to Address COVID-19 Pandemic in China: Evidence From Randomized Control Trials and Real-World Studies

**DOI:** 10.3389/fpubh.2022.917732

**Published:** 2022-07-19

**Authors:** Yaqun Fu, Jingyu Zhao, Xia Wei, Peien Han, Li Yang, Tao Ren, Siyan Zhan, Liming Li

**Affiliations:** ^1^School of Public Health, Peking University, Beijing, China; ^2^London School of Hygiene & Tropical Medicine, London, United Kingdom; ^3^Peking University Center for Public Health and Epidemic Preparedness and Response, Beijing, China

**Keywords:** effectiveness, cost-effectiveness, inactivated COVID-19 vaccine, RCT, real-world evidence, efficacy

## Abstract

**Objective:**

This study aimed to determine the efficacy, effectiveness, and cost-effectiveness of inactivated COVID-19 vaccines (CoronaVac and BBIBP-CorV) in China using existing international clinical trials and real-world evidence.

**Methods:**

Through a search of PubMed, Embase, Web of Science, and CNKI, studies investigating the effectiveness of inactivated COVID-19 vaccines were identified, and a meta-analysis was undertaken to synthesize the vaccine efficacy and effectiveness data. Moreover, a decision-analytic model was developed to estimate the cost-effectiveness of inactivated vaccines for combating the COVID-19 pandemic in the Chinese context from a societal perspective. Results of the meta-analysis, along with cost data from official websites and works of literature were used to populate the model. Sensitivity analysis was performed to test the robustness of the model results.

**Results:**

A total of 24 studies were included in the meta-analysis. In comparison to no immunization, the effectiveness of inactivated vaccine against COVID-19 infection, hospitalization, ICU admission and death were 65.18% (95% CI 62.62, 67.75), 79.10% (95% CI 71.69, 86.51), 90.46% (95% CI 89.42, 91.50), and 86.69% (95% CI 85.68, 87.70); and the efficacy against COVID-19 infection and hospitalization were 70.56% (95% CI 57.87, 83.24) and 100% (95% CI 61.72, 100). Inactivated vaccine vaccination prevented more infections, hospitalizations, ICU admissions, and deaths with lower total costs, thus was cost-saving from a societal perspective in China. Base-case analysis results were robust in the one-way sensitivity analysis, and the percentage of ICU admission or death and direct medical cost ranked the top influential factors in our models. In the probabilistic sensitivity analysis, vaccination had a 100% probability of being cost-effective.

**Conclusion:**

Inactivated vaccine is effective in preventing COVID-19 infection, hospitalization, ICU admission and avoiding COVID-19 related death, and COVID-19 vaccination program is cost-saving from societal perspective in China.

Box 1 **Summary Box**

What is already known?

- Coronavirus disease 2019 (COVID-19) has had a huge impact on the global economy and has resulted in a significant disease burden.
- The inactivated vaccines (Sinovac CoronaVac and BBIBP-CorV) have been used worldwide, and both have been validated for emergency use by the WHO. The CoronaVac is the most extensively used COVID-19 vaccine worldwide.
- Vaccination strategies have been proven to be not only cost-effective but also cost-saving in countries such as the United States, Denmark, and Turkey.

What are the new findings?

- This study is more comprehensive and contains more inactivated vaccine effectiveness data than other meta-analyses or systematic reviews, and can partially address the weakness that vaccine efficacy varies significantly across countries.
- It is the first evaluation using synthesized data pooled from randomized control trial data and real-world evidence to estimate the cost-effectiveness of inactivated vaccines compared to no vaccination in the Chinese setting.

What do the new findings imply?

- Two-dose inactivated vaccination strategy is effective and cost-saving in China.
- This study offers compelling evidence to support the free COVID-19 vaccination program in China.

## Background

Coronavirus disease 2019 (COVID-19) is a novel infectious disease caused by the severe acute respiratory syndrome coronavirus-2 (SARS-CoV-2). It is a serious crisis and a severe test for the whole world, and studies in the United States (1), the United Kingdom (2), India (3), and China (4) have shown that COVID-19 has had a huge impact on the global economy, especially the loss of productivity due to business suspension, school suspension, and business closure. As of 3 June 2022, the WHO reported about 528.82 million cumulative cases and 6.29 million cumulative deaths worldwide (5), with China reporting 2.83 million confirmed cases and 17,271 deaths (6). COVID-19 pandemic has resulted in a significant disease burden, and the virus exhibits characteristics of high infectivity, concealment, and community aggregation; most notably, outbreaks of the Omicron strain in Tianjin, Shenzhen, Shanghai, and other locations throughout China since December 2021.

To safeguard the Chinese population and economy throughout the epidemic prevention and control stage, China altered its prevention and control strategy away from medical treatment and lockdown and toward vaccination and immune barrier establishment. Since January 2021, China has offered free vaccinations, namely, inactivated vaccines (Sinovac CoronaVac and BBIBP-CorV), adenovirus-vectored vaccine (CanSino Ad5-nCoV), and protein subunit vaccine (ZF2001). BBIBP-CorV was widely used in Asia, Africa, South America, and Europe and was approved for emergency use by the WHO on 7 May 2021. Meanwhile, CoronaVac, as the most extensively used COVID-19 vaccine worldwide, was validated for emergency use by the WHO on 1 June 2021 and is currently being utilized in nations throughout Asia, America, and Eastern Europe.

Both inactivated vaccines have been proven to be effective in both Phase III clinical trials and real-world studies in many countries, such as Brazil, Chile, Turkey, Malaysia, Indonesia, and Argentina. However, the efficacy and effectiveness rates varied significantly across countries. Numerous studies investigating the efficacy of inactivated COVID-19 vaccines are now underway, although synthesized evidence of inactivated vaccine effectiveness is rare. Additionally, only a limited number of studies have been done to evaluate the cost-effectiveness of vaccination strategies, particularly the inactivated vaccine, and no studies have been identified utilizing synthesized effectiveness data. To address this gap, this study aims to summarize the effectiveness of the Chinese inactivated vaccine by combining randomized control trial (RCT) data and real-world evidence (RWE), as well as estimate the cost-effectiveness of two-dose inactivated vaccine compared to no vaccination in China from a societal perspective, in order to provide evidence for prevention and control strategy decision-making in China and globally.

## Methods

### Meta Analysis

As inactivated vaccines were the most commonly used vaccines in China, taking up to more than 90% of the market share, and Sinovac CoronaVac and BBIBP-CorV were commonly vaccinated, this study only focused on inactivated vaccines. We conducted a meta-analysis to summarize the vaccine efficacy and effectiveness (VE) of the two Chinese inactivated vaccines (CoronaVac and BBIBP-CorV) in preventing COVID-19 infection, hospitalization, ICU admission, and avoiding COVID-19-related death compared with non-vaccination groups from a societal perspective.

#### Search Strategy

This study searched English and Chinese databases, including PubMed, Embase, Web of Science, and CNKI, for studies published by 1 June 2022, using the following search terms: (effectiveness OR efficacy) AND (COVID-19 OR SARS-CoV-2) AND (vaccine OR vaccination) AND (CoronaVac OR BBIBP OR inactivated vaccine). Reference lists from relevant primary studies and review articles were also searched manually. In addition, data shared by the pharmaceutical companies was also included.

#### Study Eligibility and Selection

The target population was people susceptible to COVID-19. We focused on studies that discussed the outcomes after two doses of CoronaVac or BBIBP-CorV compared with no vaccination. The outcome measures were vaccine efficacy of RCT and the effectiveness rate of RWE against COVID-19 infection, hospitalization, ICU admission, and death. Observational studies and clinical trial studies were both included. The exclusion criteria were as follows: (a) no separate evidence for CoronaVac or BBIBP-CorV reported; (b) only geometric mean concentration (GMC) or seroconversion of neutralizing antibody data available; (c) guideline, conference, and oral report; and (d) full-text unavailable.

After removing duplicates, all initial records were screened for titles and abstracts by two independent reviewers (YF and JZ). Following this, the full texts of the shortlisted abstracts were retrieved to assess eligibility for inclusion. Any disagreement was resolved by a third reviewer (PH). VE data were extracted for relevant outcomes in pre-defined tables.

#### Statistical Analysis

Random-effects or fixed-effects models were used to pool the VE data, based on the heterogeneity between estimates (*I*^2^). The meta-analysis was conducted using the Review Manager software (version 5.4).

### Cost-Effectiveness Analysis

**Figure 1:**
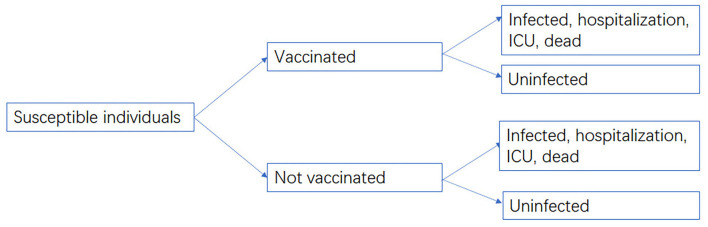
Decision-analytic model.

The cost-effectiveness between vaccination groups and non-vaccination groups were compared from a societal perspective, as the efficacy and effectiveness data of vaccines were compared with non-vaccination groups. Non-pharmaceutical interventions (NPIs) were not included in this study. A decision-analytic model was developed using TreeAge Pro 2021 (Figure 1). The cost data included vaccination cost, medical cost, and indirect cost, while the effectiveness of the vaccine against infection, hospitalization, ICU admission, and death was derived from our meta-analysis. The target population of our model was people aged over 3 years who had completed two-dose inactivated vaccination in China. The vaccinated population was 1,256.86 million, accounting for 89.15% of the total population (7). Cost data were not discounted due to the short time period.

#### Natural Infection Rate and Percentage of Each Status

There was a paucity of data on the natural infection rate of unvaccinated people. Only an RCT of BBIBP-CorV in UAE and Bahrain (8) and a real-world study of CoronaVac in Chile (9) reported the natural infection rate. The natural infection rate from the RWE research in Chile was adopted since it encompassed approximately 80% of its population and was more representative. Furthermore, the declining exponential approximation of life expectancy (DEALE) method (10) was applied to convert the incidence in a cohort (per person-year) to the probability of infection in 1 year. The calculation of 1-year infection probability is *p* = 1−*e*^−*r*^. Here, *p* is the 1-year infection probability and *r* (person-year) is the incidence rate.

As for other transition probabilities, the transition pattern between infection, hospitalization, and ICU or death was obtained from the patient proportion in China (11).

#### Costs

Vaccination cost, medical cost, and indirect cost were included in this study. Due to the lack of a systematic review of the economic burden of COVID-19 in China, medical and indirect cost data mostly came from real-world sources, such as the literature of the Wuhan pandemic. Specifically, vaccination costs comprised vaccine procurement, cold-chain transportation, refrigeration, and administration, whereas medical costs covered diagnosis, treatment, hospitalization, and care expenditures associated with sickness. Although the expenses of COVID-19 vaccination and treatment are fully covered by basic medical insurance and the Ministry of Finance, this analysis included all the vaccination and medical costs incurred through the health system. Additionally, the productivity loss due to illness or premature death was included as the indirect cost from a societal perspective.

The cost of each dose of inactivated vaccine came from the lowest global purchase price published by the WHO (12). The transportation cost of vaccine was assumed to be 6% of the purchase cost (13), the refrigeration cost was calculated based on WHO recommendation, and the vaccine administration fee (injection service fee) was US$1.55 (14) (US$1 = ¥6.449).

Medical costs increased as disease severity grew from mild to severe to critical, according to data from an economic impact study of COVID-19 during the early stages of the Wuhan outbreak, and were US$868.51, US$8,210.73, and US$23,467.82 for infection, hospitalization, and ICU or death, respectively (4). Productivity losses were calculated based on the proportion of the labor force and the average daily salary (15).

#### Base Case Analysis

The outcome of the cost-effectiveness analysis was the number of infection cases, hospitalization, ICU admissions, and deaths avoided. The incremental cost-effectiveness ratio (ICER) was calculated to determine whether vaccination was cost-effective or not, and the threshold was set at the gross domestic product (GDP) per capita of China in 2021 (US$ 12,556.37) (15).

#### Sensitivity Analysis

One-way sensitivity analysis and probabilistic sensitivity analysis (PSA) were performed for the base case results from a societal perspective. The range of each parameter was derived from the published literature or our meta-analysis. The results were shown as tornado diagrams. As for the PSA, Monte Carlo simulation (*N* = 1,000 iterations) was used to assess the effects of changing multiple parameters simultaneously. Gamma and Dirichlet distributions were assigned to costs, with Beta distributions to vaccine efficacy and proportions of different infection severities. The PSA results were presented as cost-effectiveness acceptability curves.

### Patient and Public Involvement

Not applicable.

## Results

### Meta Analysis of Vaccine Effectiveness

**Figure 2:**
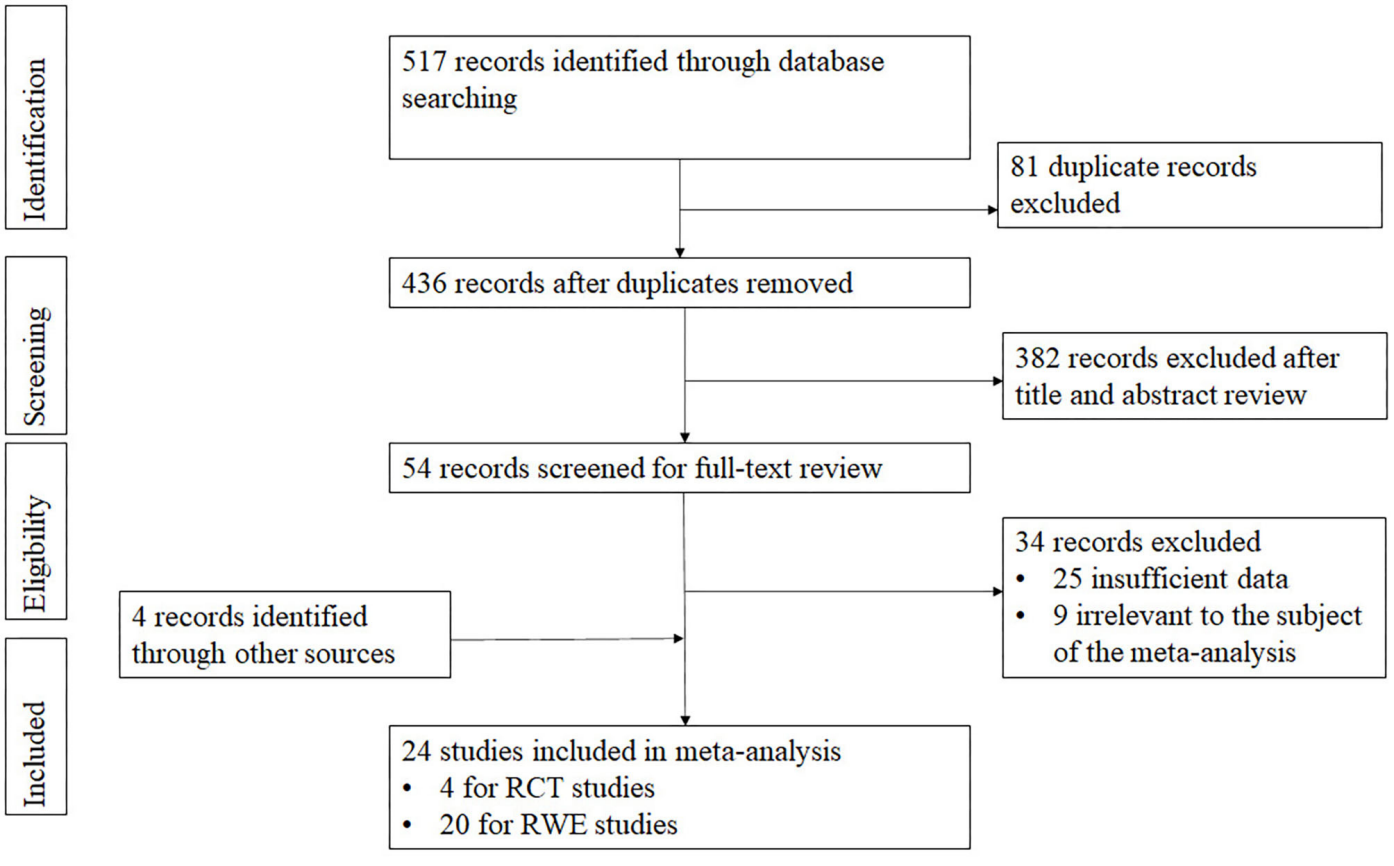
PRISMA flowchart for review of the study selection.

A total of 517 articles from four databases were extracted. Of them, 81 articles of duplication were excluded. Following a review of the title and abstract, we excluded 382 records not related to inactivated vaccine or only mentioning GMC or seroconversion of neutralizing antibody. Among the 54 studies under full-text review, 34 studies were excluded (Figure 2). Ultimately, with four records identified through other sources included, the meta-analysis comprised 24 eligible studies finally, including four phase 3 studies (8, 16–18) and 20 real-world studies (9, 19–35) (including 2 RWE shared by company). The studies mentioned virus strains were as follows: four of Alpha (22, 23, 31, 32), one of Beta (31), three of Gamma (19, 22, 27), five of Delta (20, 21, 25, 28, 29), one of Omicron (30), while the others did not mention the virus type. The majority of the included studies focused on adults, one focused on children aged 3–5 years (30), and three focused on the elderly aged over 60 years (22, 23, 33, 35).

**Figure 3:**
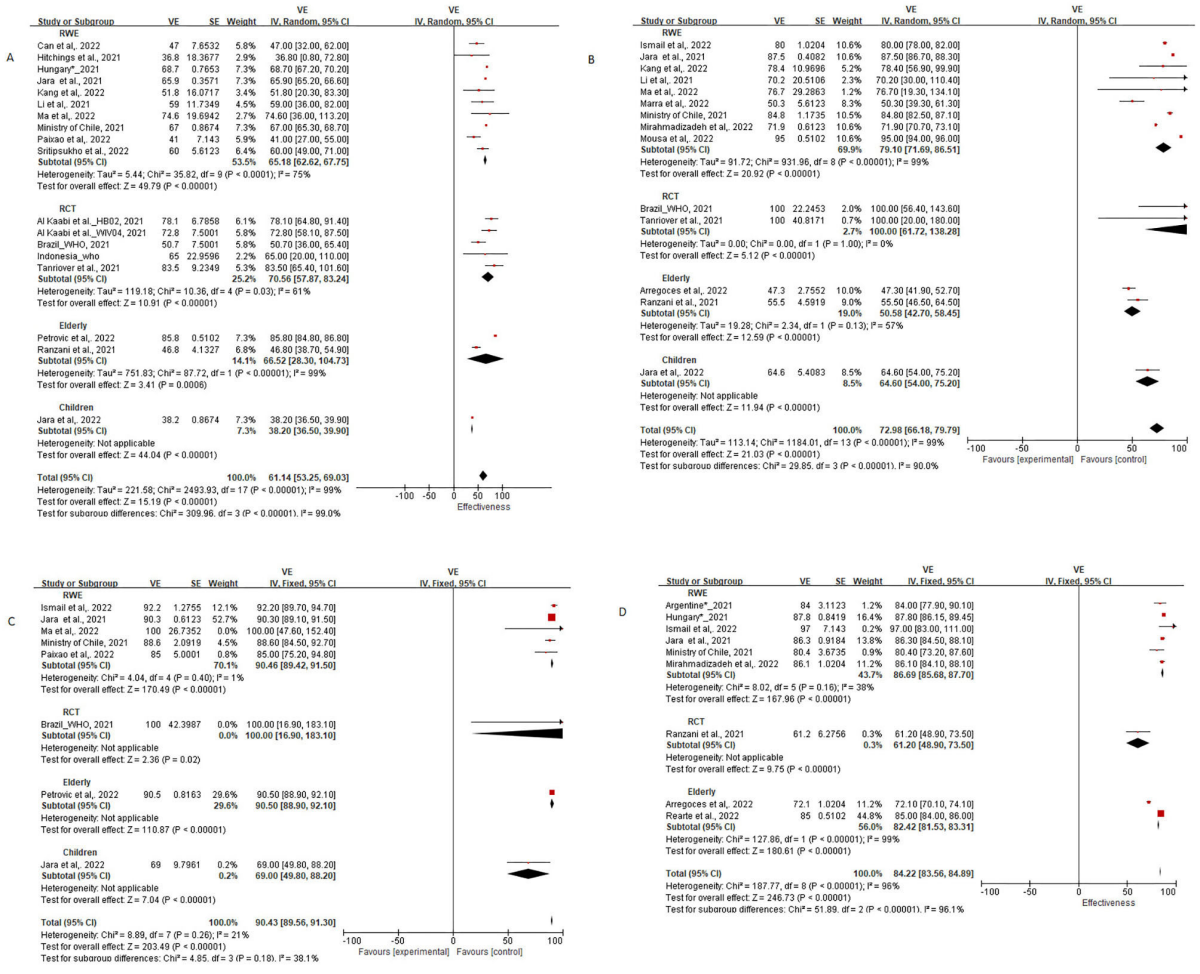
Forest plots for the vaccine effectiveness (VE) of inactivated vaccine. **(A)** Forest plots against COVID-19 infection, **(B)** Forest plots against COVID-19 hospitalization, **(C)** Forest plots against COVID-19 ICU admission, **(D)** Forest plots against COVID-19 related death. ^*^Internal data shared by company.

In the meta-analysis fixed-effects model was used when *I*^2^ <50%, and the random-effects model was used when the *I*^2^ > 50% to pool the VE data with 95% confidence intervals (CIs). Among the included articles, RCT evidence from Turkey (16), Brazil (18, 36), Indonesia (17), and the United Arab Emirates and Bahrain (8); RWE data from Chile (9, 30, 34), Brazil (19, 24, 27, 35), Argentina (22), Thailand (21), Serbia (23), United Arab Emirates (25, 31), Iran (26), China (20, 28, 29), Colombia (33), Hungary, and Turkey (32) were included. As the heterogeneity among RCT and RWE was huge, and *I*^2^ was consistently higher than 90%, this study was not able to pool efficacy and effectiveness data together; therefore, we have done subgroup analysis for RCT and RWE studies of adult, and for the elderly and children. The effectiveness of inactivated vaccine in real-world settings of adults against COVID-19 infection, hospitalization, ICU admission, and death were 65.18% (95% CI: 62.62, 67.75), 79.10% (95% CI: 71.69, 86.51), 90.46% (95% CI: 89.42, 91.50), and 86.69% (95% CI: 85.68, 87.70), while the efficacy in clinical trials against COVID-19 infection and hospitalization were 70.56% (95% CI: 57.87, 83.24) and 100% (95% CI: 61.72, 100; Table 1). The forest plots are shown in Figure 3.

**Table 1:** Model parameters.

**Item**	**Parameter**	**Value**	Lower^*^	Upper^*^	**Distribution**	**Data source**
Vaccination cost per dose (USD)	Acquisition	4.00	4	5.5	Gamma	WHO (12)
	Cold-chain freight	0.24	/	/		Chen et al. (13)
	Refrigerator storage	0.18	/	/		Jiang et al. (10)
	Administration	1.55	/	/		National medical insurance bureau (14)
Direct medical cost (USD)	Infection/Non-severe	868.51	613.58	1,788.03	Dirichlet	Jin et al. (4); Zhao et al. (11)
	Hospitalization/Severe	8,210.73	5,800.74	16,905.10	Dirichlet	
	ICU/Critical/death	23,467.82	16,710.96	48,700.88	Dirichlet	
Percentage of each status	Infection	81.50%	60%	90%	Beta	Jin et al. (4); Zhao et al. (11)
	Hospitalization	13.80%	10%	20%	Beta	
	ICU /death	4.70%	1%	10%	Beta	
Work time loss/day	Infection	37	/	/		Jin et al. (4); Zhao et al. (11)
	Hospitalization	41	/	/		
	ICU	42	/	/		
	Death	44	/	/		
Average salary per day (USD)		42.18	31.79	75.36	Gamma	National bureau of statistics (15)
Labor force participation	Aged 18–60	60.90%	/	/		The seventh national population census (37)
Nature rate	Infection rate	0.10430	/	/		Calculated
	Mortality rate	0.00164	/	/		Calculated
Vaccine effectiveness of adult (%)	Against infection	65.18%	62.62	67.75	Beta	Meta-analysis
	Against hospitalization	79.10	71.69	86.51	Beta	Meta-analysis
	Against ICU admission	90.46%	89.42	91.50	Beta	Meta-analysis
	Against death	86.69%	85.68	87.70	Beta	Meta-analysis
Vaccine effectiveness of elderly (%)	Against infection	66.52	28.30	100.00	Beta	Meta-analysis
	Against hospitalization	50.58	42.70	58.45	Beta	Meta-analysis
	Against death	82.42	81.53	83.31	Beta	Meta-analysis
Vaccine efficacy (%)	Against infection	70.56	57.87	83.24	Beta	Meta-analysis
	Against hospitalization	100.00	61.72	100.00	Beta	Meta-analysis
No. of population	Vaccinated with two doses (million)	1,256.86	/	/		State council (7)
Threshold (USD)	GDP per capital	12556.37	/	/		National bureau of statistics (15)

* *Upper and lower bound are obtained from published literature and meta-analysis*.

### Cost-Effectiveness Analysis

#### Base Case Analysis

The cost-effectiveness analysis showed that although the RCT and RWE data cannot be pooled together, two-dose inactivated vaccination were consistently cost-saving in preventing COVID-19 infection, hospitalization, ICU admission, and avoiding COVID-19-related death. The ICER were calculated using RWE data of adults and were US$-3,739 per preinfected case avoided, US$-12,364 per severe case avoided, US$-21,853 per ICU case avoided, and US$-16,197 pre death avoided (Table 2). The same trend was seen while using RCT data. To summarize, vaccination strategy is consistently cost-saving and cost-effective in the Chinese setting from a societal perspective, and it is necessary to continue adhering to vaccination strategy to protect population health.

**Table 2:** Cost-effectiveness of inactivated vaccine against COVID-19 infection, hospitalization, ICU admission, and death.

**Vaccine effectiveness**	**Group**	**Cost (US$)**	**Effectiveness (No. of events)**	**Incremental cost** **(US$)**	**Incremental effectiveness (No. of events)**	**ICER** **(US$/event)**
Against infection (RCT)	Two doses	168,553,644,312	39,166,629	−352,229,625,481	93,872,193	−3,752
	Non-vaccination	520,783,269,793	133,038,822	/	/	/
Against infection (RWE)	Two doses	196,571,784,227	46,324,118	−324,211,485,566	86,714,704	−3,739
	Non-vaccination	520,783,269,793	133,038,822	/	/	/
Against hospitalization (RCT)	Two doses	15,235,049,685	0	−308,329,723,032	24,612,182	−12,528
	Non-vaccination	323,564,772,717	24,612,182	/	/	/
Against hospitalization (RWE)	Two doses	82,860,087,183	5,143,946	−240,704,685,534	19,468,236	−12,364
	Non-vaccination	323,564,772,717	24,612,182	/	/	/
Against ICU admission (RWE)	Two doses	29,877,607,019	596,519	−123,608,318,918	5,656,305	−21,853
	Non-vaccination	153,485,925,937	6,252,825	/	/	/
Against death (RWE)	Two doses	22,083,877,846	278,430	−29,372,381,818	1,813,456	−16,197
	Non-vaccination	51,456,259,664	2,091,886	/	/	/

#### Sensitivity Analysis

**Figure 4:**
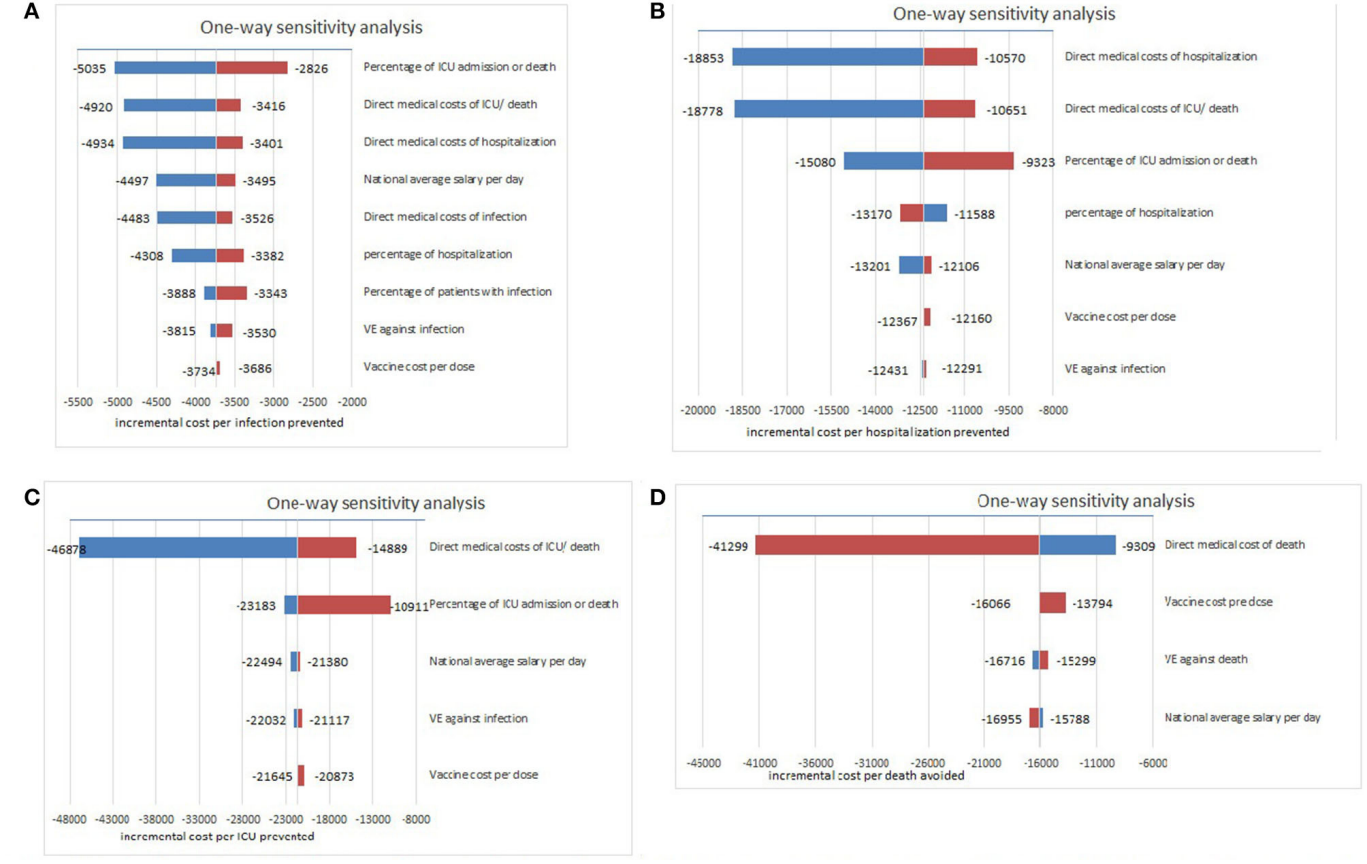
One-way sensitivity analyses for the model on ICER (US$/event). **(A)** Sensitivity analysis for model against COVID-19 infection, **(B)** Sensitivity analysis for model against COVID-19 hospitalization, **(C)** Sensitivity analysis for model against COVID-19 ICU admission, **(D)** Sensitivity analysis for model against COVID-19 related death.

The model results were found to be robust in the one-way sensitivity analysis, indicating that two-dose inactivated vaccine strategy was always cost-saving. First, for infection prevention, percentage of ICU admission or death, direct medical costs of ICU/death, and direct medical costs of hospitalization for critical cases were the most influential parameters (Figure 4A). Second, direct medical costs of hospitalization, ICU/death, and percentage of ICU admission or death influenced the model most when considering hospitalization prevention (Figure 4B). Third, for ICU prevention, direct medical costs of ICU/death, and percentage of ICU admission, and national average salary per day were the most influential factors (Figure 4C). Finally, for death prevention, direct medical costs of death affected the model most (Figure 4D), indicating that with the decrease in medical costs and the declining severity of disease, vaccination strategies could be more cost-saving from a societal perspective, and more effective treatment methods should be adopted.

**Figure 5:**
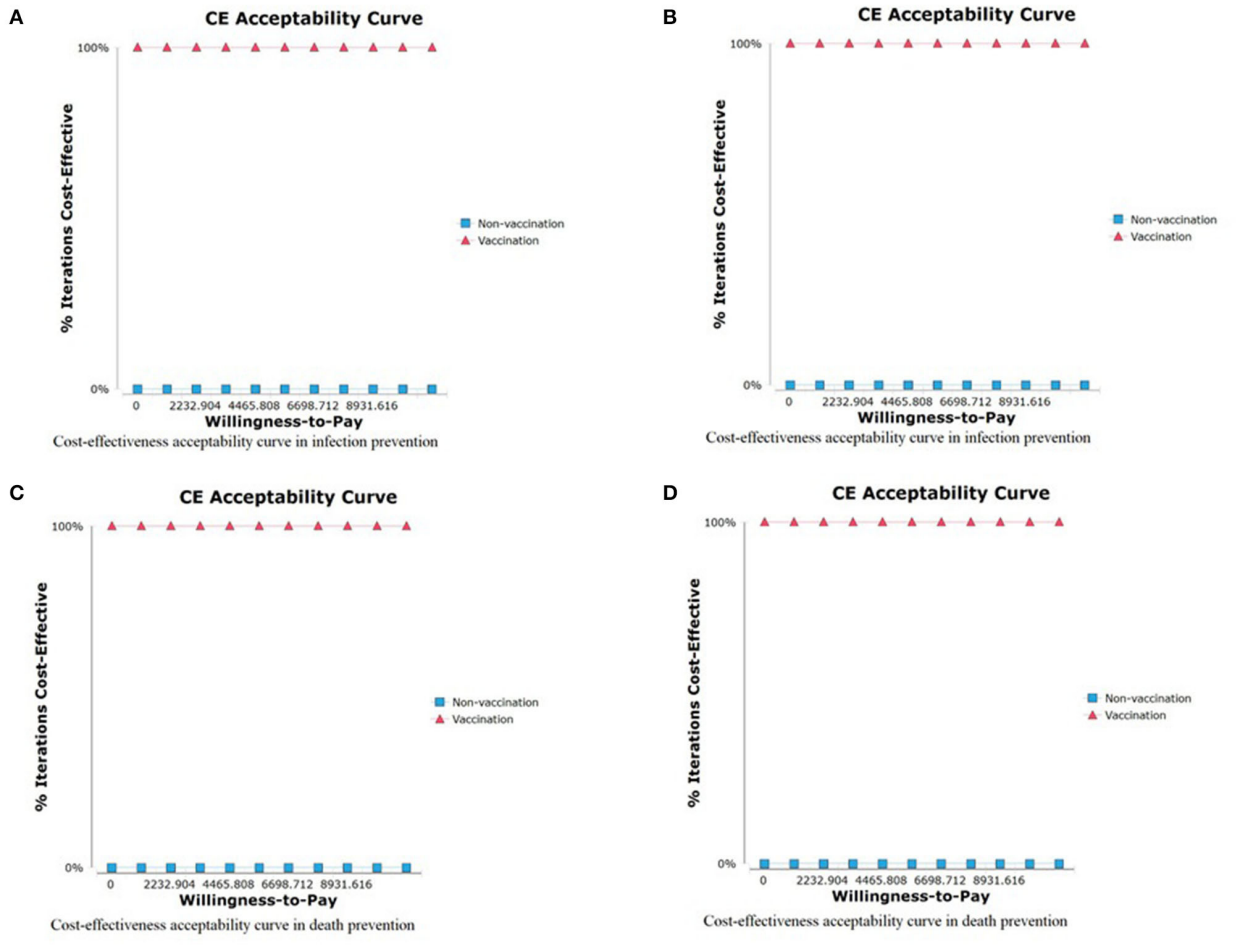
Cost-effectiveness acceptability curve. **(A)** CEA curve of COVID-19 infection prevention, **(B)** CEA curve of COVID-19 hospitalization prevention, **(C)** CEA curve of COVID-19 ICU admission prevention, and **(D)** CEA curve of COVID-19 related death COVID-19.

In the PSA, the probability of the vaccination strategy being cost-effective was 100% in preventing infection, hospitalization, ICU admission, and avoiding death, regardless of the willingness to pay (Figure 5).

## Discussion

This study summarized the inactivated COVID-19 vaccine efficacy and effectiveness evidence from 24 RCT and real-world studies and is, to our knowledge, more extensive and contained other meta-analyses or systematic reviews on inactivated vaccine (38–42). As the pandemic control strategy, study population, and sample size vary among countries, we synthesized both RCT and RWE data to generate more representative evidence. Our findings corroborate prior analyses of the literature, and the pooled VE indicated that inactivated vaccine is highly effective in preventing COVID-19-related infections, hospitalization, ICU admission, and death. The cost-effectiveness analysis showed that the vaccination strategy was cost-saving compared with non-vaccination in China.

Our results were consistent with previous cost-effectiveness studies and can improve their findings as these studies were done at the early stage of the COVID-19 pandemic with insufficient data on the efficacy and vaccine cost. Specifically, evidence from high-income countries showed that in general vaccination was cost-saving (43–45) and cost-effective (46, 47), and the whole society can benefit from vaccination even when the coverage rate was at 60% (44). As for low- and middle-income countries, vaccination was cost-saving from a societal perspective (10, 48). A Ukraine study illustrated that the priority strategy for older adults was more cost-effective when vaccine supply was insufficient (49). However, the majority of these studies used a vaccine with hypothetical efficacy, coverage rate, and cost data, and none of the studies mentioned the type of vaccine. There is a limited number of studies focusing on a specific type of vaccine, such as a Taiwan study evaluating mRNA vaccines (BNT162b2 and mRNA-1273) and adenovirus vaccines (AZD1222) (50), and a cross-country/region study examining inactivated vaccines (10), and the results are consistent with previous findings. In conclusion, there is limited evidence on the economic evaluation of inactivated vaccines, especially in low- and middle-income settings.

China has offered free vaccination to cover the whole population, and the first step was to cover people aged 18–59 years, which gradually extended to those aged over 60 years in April 2021, to adolescents aged 12–17 years in July 2021, and to children aged 3–11 years in November 2021. Previous study focusing on the cost-effectiveness of inactivated vaccine suggested that mass vaccination program should be encouraged (10), and our findings indicated that the high vaccination coverage rate can also benefit Chinese society. Moreover, due to the fact that both of the inactivated vaccines have been validated for emergency use by the WHO at a relatively low price, the results of this study can be used for countries or regions where vaccination coverage is high.

By strictly adhering to the containment strategy to safeguard its population, as detailed in the pamphlet Fighting COVID-19 China in Action (51), the pandemic has been effectively controlled in China mainland. The cost-effectiveness results in our study can provide compelling evidence to support the current vaccination strategy as well as evidence for subsequent decision-making, particularly in health sectors.

When the long-term effects of virus strains in human, the shock to the medical system (52) or health insurance system (53), and the uncertainty impacts on social stability are considered, vaccination is likely to be more cost-effective than the non-vaccination strategies, and the ICER could be much lower than our estimates. In addition, as the cost of treatment affected the model most in the one-way sensitivity analysis, encouraging asymptomatic patients and mild patients to stay at home or be treated at cabin hospitals instead of crowding into hospitals could save costs from a societal perspective and save medical resources. Therefore, it is necessary to continue adhering to the vaccination strategy to protect population health and maintain social stability and economic development.

Except for the free COVID-19 vaccination program, such as mask wearing, social distancing, quarantine, contact tracing, business closure, and lockdown, are adopted by the Chinese government. Additionally, the government also takes measures to release the financial burden of its population by capping nucleic acid testing costs at US$6.20 per individual test and US$1.55 per mixed test (54), increasing labor productivity by encouraging telecommuting, and encouraging small and medium-sized enterprises by reducing the taxation and increasing subsidy (55).

It is also worth noting that further studies on the exploration of the most cost-effective combination of vaccines and other NPIs strategies in real-world scenarios, the reduction of unnecessary lockdown and containment policies, and maximizing the smooth operation of society and economic development are needed.

This study has some limitations. First, statistics on medical costs were gathered during the early stage of the COVID-19 outbreak in Wuhan. Medical cost data may alter as the pandemic progresses, diagnosis and treatment guidelines improve, and the infectivity and pathogenicity of the virus change. However, this study contains the most comprehensive and robust data on medical costs. Second, the VE are from overseas studies and may not be representative for the Chinese population; however, due to the limited number of trials conducted and the small number of COVID-19 cases in China, the results of this study remain the most reliable estimate. Third, the efficacy and effectiveness data were collected throughout the pandemic, so the protection rate cannot be simply extrapolated to Delta or Omicron strain virus; however, under the Delta and Omicron virus pandemic, attention should be paid to the effectiveness of booster vaccination after the initial immunization procedure. Finally, neither the influence of NPIs nor the effects of pandemic control, economic sustainability, and social stability were considered in this study. In light of the long-term effects, this study may underestimate the importance of immunization.

## Conclusion

The inactivated vaccine is effective in preventing COVID-19 infection, hospitalization, ICU admission, and avoiding COVID-19-related death, and free COVID-19 vaccination program is consistently cost-saving from both health system and societal perspective in China. Therefore, the entire Chinese population should receive two doses of inactivated vaccine. Moreover, further studies on booster vaccination are necessary to determine the most cost-effective and long-lasting approach of COVID-19 prevention and control.

## Data Availability Statement

The modeling presented in this study is parameterized by data from public sources.

## Author Contributions

LY conceived this analysis article. YF and JZ collected the evidence and drafted the manuscript. LY, XW, and PH reviewed and edited the manuscript. LY, TR, SZ, and LL coordinated the overall process. All authors approved the final manuscript.

## Funding

This study was supported by a grant from the National Natural Science Foundation of China [72174010], the Beijing Natural Science Foundation [M22033], and the Capital Health Research and Development of Special Fund [2021-1G-4091].

## Conflict of Interest

The authors declare that the research was conducted in the absence of any commercial or financial relationships that could be construed as a potential conflict of interest.

## Publisher's Note

All claims expressed in this article are solely those of the authors and do not necessarily represent those of their affiliated organizations, or those of the publisher, the editors and the reviewers. Any product that may be evaluated in this article, or claim that may be made by its manufacturer, is not guaranteed or endorsed by the publisher.

## Abbreviations

COVID-19: Coronavirus disease 2019
CIs: Confidence intervals
DEALE: Declining exponential approximation of life expectancy
GMC: Geometric mean concentration
ICERs: Incremental cost-effectiveness ratios
NPIs: Non-pharmaceutical interventions
RCT: Randomized control trial
RWE: Real-world evidence
SARS-CoV-2: Severe acute respiratory syndrome coronavirus-2
VE: Vaccine effectiveness.
